# Tmem119 expression is downregulated in a subset of brain metastasis-associated microglia

**DOI:** 10.1186/s12868-024-00846-3

**Published:** 2024-02-02

**Authors:** Weili Ma, Jack Oswald, Angela Rios Angulo, Qing Chen

**Affiliations:** Immunology, Metastasis and Microenvironment Program, The Wistar Institute, 3601 Spruce Street, 19104 Philadelphia, PA USA

**Keywords:** Tmem119, Microglia, Cancer, Metastasis

## Abstract

Under pathological conditions, the immune-specialized brain microenvironment contains both resident microglia and bone marrow-derived myeloid cells recruited from peripheral circulation. Due to largely overlapping phenotypic similarities between these ontogenically distinct myeloid populations, studying their individual functions in central nervous system diseases has been challenging. Recently, transmembrane protein 119 (Tmem119) has been reported as a marker for resident microglia which is not expressed by bone marrow-derived myeloid cells. However, several studies have reported the loss or reduction of Tmem119 expression in pathologically activated microglia. Here, we examined whether Tmem119 could be used as a robust marker to identify brain metastasis-associated microglia. In addition, we also compared Tmem119 expression of primary microglia to the immortalized microglia-like BV2 cell line and characterized expression changes after LPS treatment. Lastly, we used a commercially available transgenic mouse line (Tmem119-eGFP) to compare Tmem119 expression patterns to the traditional antibody-based detection methods. Our results indicate that brain metastasis-associated microglia have reduced Tmem119 gene and protein expression.

## Background

Microglia are the major resident immune cells of the central nervous system (CNS) which are activated in response to injury and disease [1]. Under pathological conditions, however, there is also an influx of bone marrow-derived myeloid cells from the peripheral circulation [2]. These different myeloid cells have been observed in both primary and metastatic brain tumors, though whether they have shared function in disease progression remains unclear [3]. Next-Generation Sequencing studies have elucidated transcriptomic differences between these distinct myeloid cells, suggesting potentially differing functions during intracranial tumor progression [4–6].

While microglia and bone marrow-derived macrophages are ontogenically distinct [7], they share many overlapping phenotypical markers, making experimental investigation challenging. Thus, there has been significant effort put forth into defining robust distinguishing markers. Recently, transmembrane protein 119 (Tmem119) has been shown to be a promising marker which labels over 90% of brain-resident microglia, but not macrophages [8]. It is reported to have stable expression in vivo and can be used to identify resident microglia in murine models of sciatic nerve injury, optic nerve crush, and lipopolysaccharide (LPS) treatment [8]. Furthermore, TMEM119 expression could be detected in human post-mortem samples of Alzheimer’s disease [9] and traumatic brain injury [10].

Although Tmem119 appears to be a robust homeostatic microglia marker, several studies have reported fluctuating expression under various pathological conditions. Tmem119 immunoreactivity was found to significantly decrease in mouse models of traumatic brain injury [11] and ischemic stroke [12]. In human clinical samples, Tmem119 expression was reduced in the white matter lesions but not gray matter lesions of multiple sclerosis [13]. While Tmem119 is detectable in Alzheimer’s disease, its expression was significantly reduced [14]. Thus, microglia activation state may affect the expression of Tmem119. Furthermore, it is currently unknown whether a reduced Tmem119 expression may limit its use in brain tumor models where there is a large fraction of peripheral myeloid cells in the microenvironment.

In this study, we sought to investigate Tmem119 expression patterns in brain metastasis-associated microglia. We compared detection of Tmem119 using commercially available antibodies and the Tmem119-eGFP transgenic mice [15]. We also investigated Tmem119 expression patterns in BV2 cells, a commonly used immortalized murine microglia cell line. Finally, we used a murine model of breast cancer brain metastasis (E0771-BrM) to assess the reliability of Tmem119 as a marker to distinguish tumor-associated microglia from peripheral myeloid cells.

## Results

### Brain region dependent Tmem119 expression in primary microglia

**Fig. 1:**
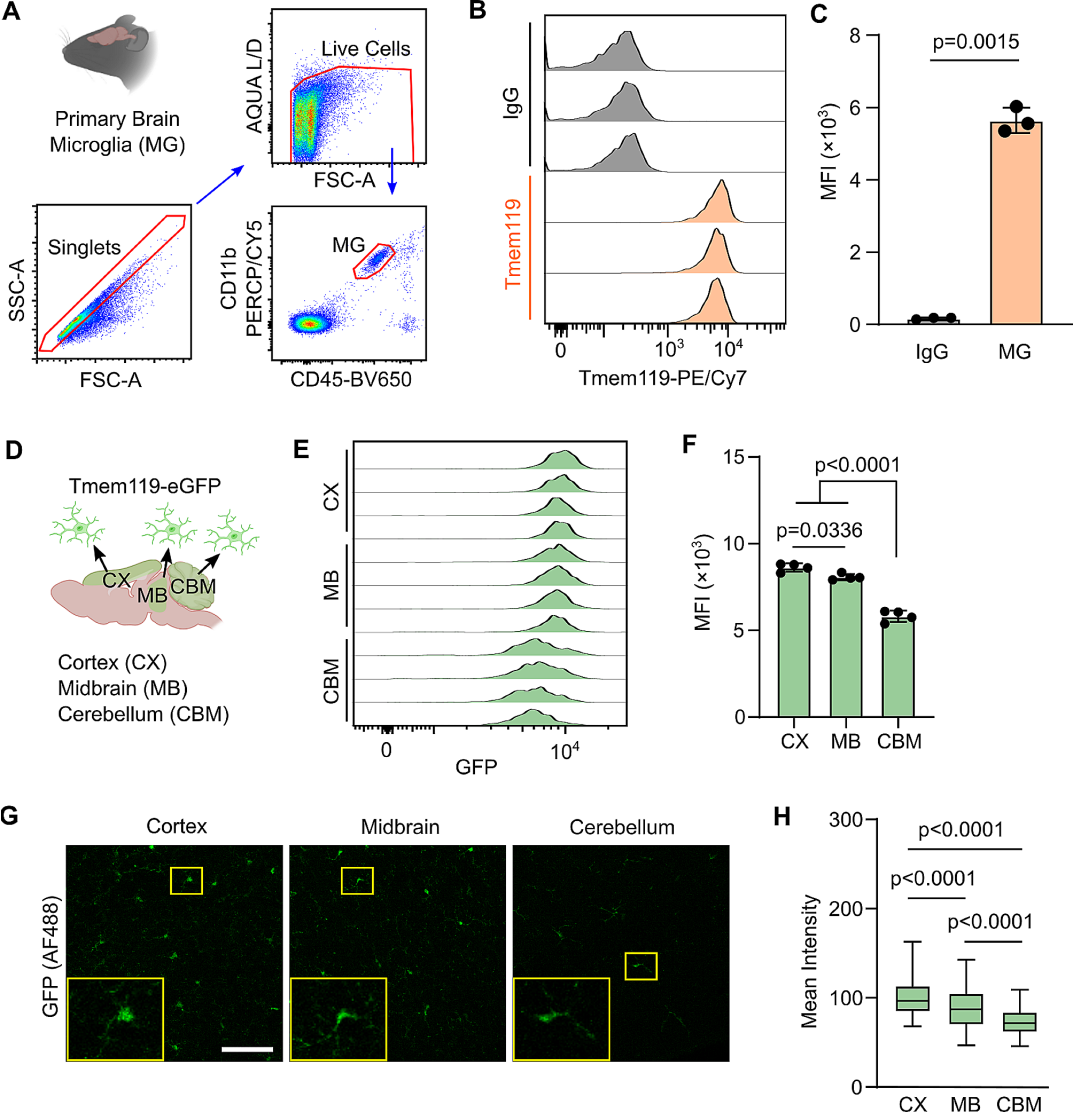
Basal Tmem119 expression in primary murine microglia (MG). (**A**) Gating strategy for primary microglia (CD45^int^ CD11b^pos^) from mouse brains. (**B**) Representative flow cytometry histograms of Tmem119-PE/CY7 compared to isotype controls. (**C**) Quantification of Tmem119-PE/CY7 MFI. *P* value is the result of unpaired two-tailed T-test. Data are presented as mean ± S.D. *N* = 3 mice. (**D**) Microglia from cortex (CX), midbrain (MB), and cerebellum (CBM) of naïve Tmem119-eGFP transgenic mice were compared for Tmem119 expression levels. (**E**) Flow cytometry histograms of Tmem119-eGFP from microglia in various brain regions. (**F**) Quantification of Tmem119-eGFP MFI. *P* values are results of One-way ANOVA with Tukey HSD. Data are presented as mean ± S.D. *N* = 4 mice. (**G**) Representative immunofluorescence microscopy images of Tmem119-eGFP microglia in different brain regions. Scale bar = 80 μm. Insets (yellow borders): Enlarged area showing single microglia. (**H**) Quantification of GFP-staining intensity in microglia from different brain regions. *P* values are the result of one-way ANOVA with Tukey HSD. *N* = 285 CX microglia, *N* = 162 MB microglia, and *N* = 52 CBM microglia from 3 different sections per region

First, we confirmed high expression of Tmem119 by homeostatic microglia using flow cytometry on single cell suspensions prepared from whole mouse brains (Fig. 1A). In these experiments, microglia were identified by CD45^int^CD11b^+^ staining. A high signal from Tmem119 was detected using a commercially available antibody compared to isotype controls (Fig. 1B and C). Since microglia are known to have phenotypic heterogeneity within different brain regions [16, 17], we investigated whether Tmem119 expression could be region dependent. We used the commercially available Tmem119-eGFP transgenic mice (JAX strain 031823) [15] to compare microglia Tmem119 expression in the cortex (CX), midbrain (MB), and cerebellum (CBM) (Fig. 1D). After quantifying the mean fluorescence intensity (MFI), CX microglia was found to have significantly higher Tmem119 expression compared to MB microglia, and CBM microglia had the lowest Tmem119 expression overall (Fig. 1E and F). We further validated these findings by immunofluorescence staining of brains from Tmem119-eGFP mice (Fig. 1G). Again, quantification of mean staining intensity in the different brain regions revealed a significant difference in Tmem119 expression. In agreement with the flow cytometry data, CX microglia had the brightest Tmem119 staining, MB microglia had intermediate staining intensity, and CBM microglia had the lowest staining intensity (Fig. 1H). We conclude that Tmem119 is a strong marker for homeostatic microglia, but its expression can vary by brain region.

### Loss of Tmem119 expression in activated microglia

**Fig. 2:**
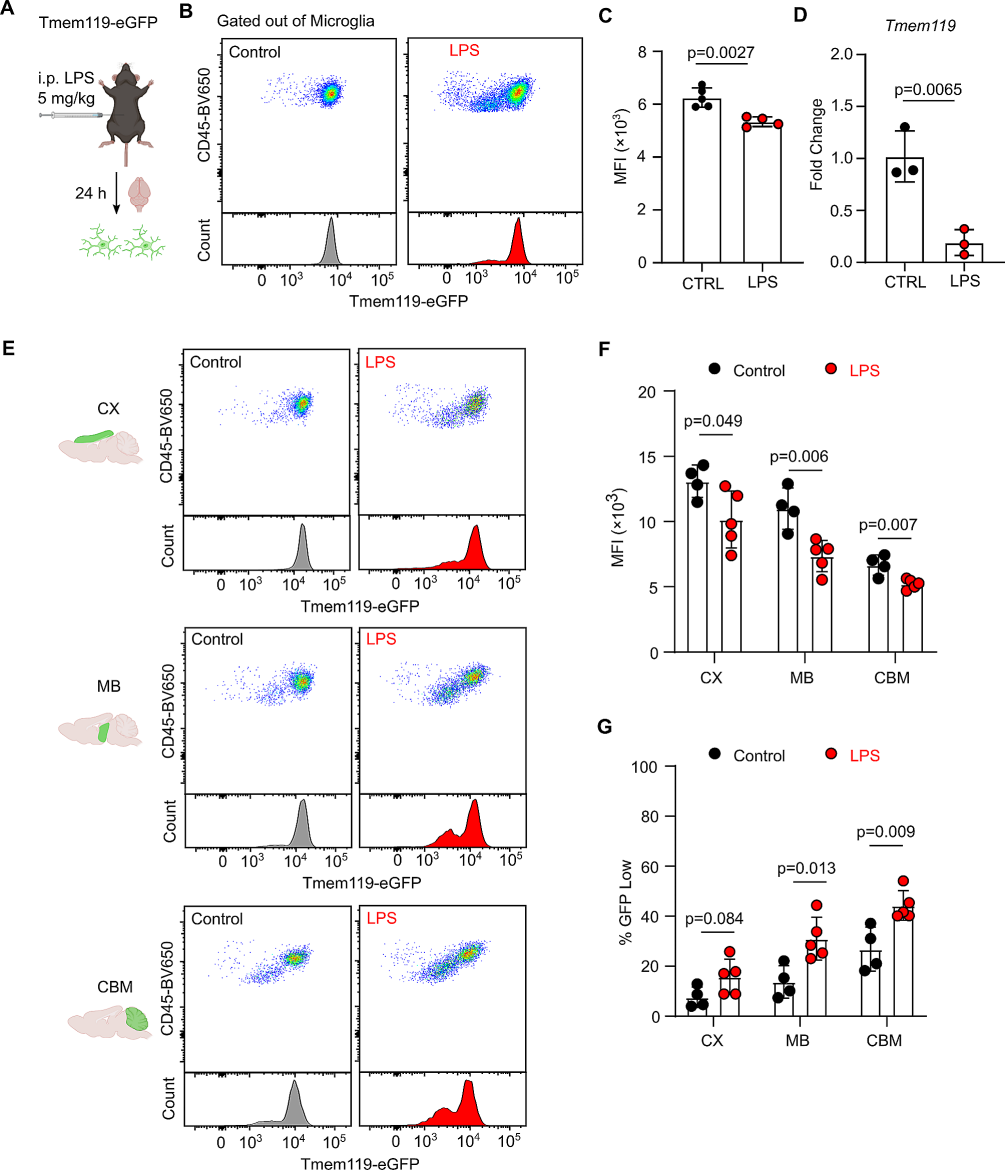
Changes in microglia Tmem119 expression after LPS treatment. (**A**) Tmem119-eGFP mice were treated with 5 mg/kg LPS for 24 h prior to analysis. (**B**) Representative flow cytometry dot plot and histogram of Tmem119-eGFP signal in control and LPS groups. (**C**) Quantification of Tmem119-eGFP MFI. *P* value is the result of unpaired two-tailed T-test. *N* = 4 mice per group. (**D**) RT-qPCR results of *Tmem119* expression in microglia sorted from brains of control and LPS groups. *P* value is the result of unpaired two-tailed T-test. *N* = 3 mice per group. (**E**) Representative flow cytometry dot plot and histogram of Tmem119-eGFP in microglia from various brain regions in control and LPS groups. (**F**) Quantification of microglia Tmem119-eGFP MFI from various brain regions in control and LPS groups. *P* values are the result of unpaired two-tailed T-tests. *N* = 4 control and 5 LPS-treated mice. (**G**) Quantification of GFP^low^ microglia population in control and LPS groups from various brain regions. *P* values are the result of unpaired two-tailed T-tests. *N* = 4 control and 5 LPS-treated mice

Next, we sought to determine changes in Tmem119 gene and protein expression in activated microglia. We induced microglia activation with lipopolysaccharide (LPS), a strong inflammatory stimulant which can readily cross the blood-brain barrier [18]. Tmem119-eGFP mice received an intraperitoneal injection of 5 mg/kg LPS 24 h before euthanasia (Fig. 2A). After LPS treatment, we detected a significant reduction in Tmem119 expression by primary microglia (Fig. 2B and C). The loss of Tmem119 was also observed at the gene expression level using RT-qPCR (Fig. 2D). Although the RT-qPCR results indicate a general loss of *Tmem119*, the flow cytometry profiles suggest that only a subset of microglia have reduced Tmem119 expression after LPS treatment. We examined whether microglia from different brains regions have different patterns of Tmem119 expression after LPS treatment. Using flow cytometry, we detected a significant reduction in Tmem119-eGFP MFI after LPS treatment (Fig. 2E and F). MB microglia had the largest reduction in Tmem119 expression after LPS treatment (1.5-fold), while CX and CBM microglia had a 1.3-fold decrease. In all brain regions tested, we again observed that only a subset of microglia reduced Tmem119 expression. Quantification of GFP^low^ microglia after LPS treatment shows a significant increase in this population among all brain regions (Fig. 2G). Consistent with the MFI results, MB showed the highest fold increase in GFP^low^ microglia (2.3-fold) compared to CX (2-fold) and CBM (1.7-fold). Thus, we conclude that Tmem119 expression is reduced on activated microglia. Furthermore, this expression appears to be more strongly affected in MB microglia.

### BV2 cells do not recapitulate Tmem119 expression patterns of primary microglia

**Fig. 3:**
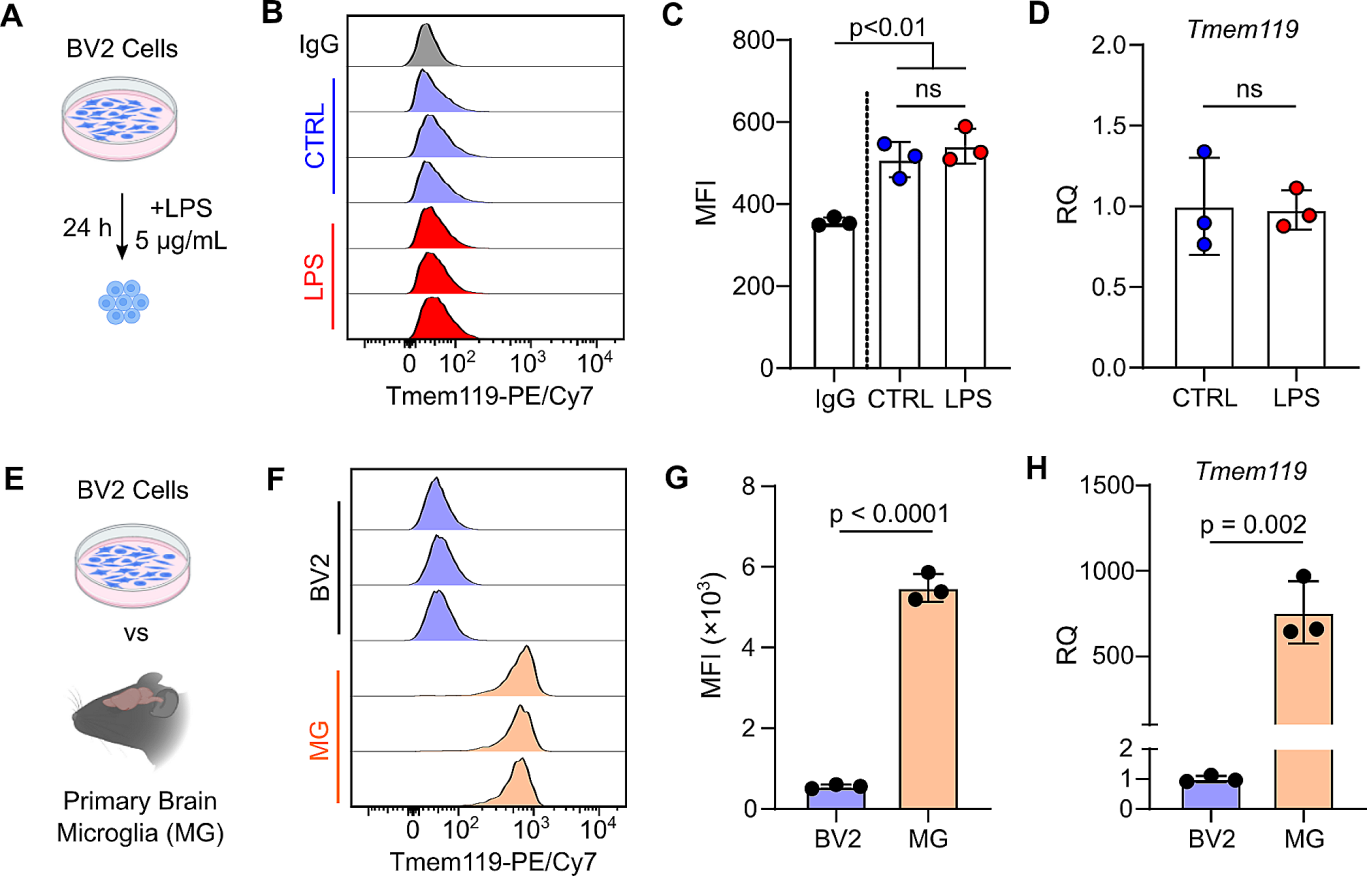
BV2 cells do not recapitulate Tmem119-expression patterns of primary microglia. (**A**) BV2 cells were treated with 5 μg/mL LPS for 24 h and collected for experiments. (**B**) Flow cytometry histograms of Tmem119-PE/Cy7 on BV2 cells in control and LPS groups. (**C**) Quantification of BV2 Tmem119-PE/Cy7 MFI in control and LPS groups. *P* values are the result of one-way ANOVA with Tukey HSD. *N* = 3 replicates of BV2 cells. (**D**) RT-qPCR of *Tmem119* expression in BV2 cells in control and LPS groups. *P* value is the result of unpaired two-tailed T-test. *N =* 3 replicates of BV2 cells. (**E**) Comparison of Tmem119 expression in BV2 cells and primary microglia. (**F**) Flow cytometry histogram of Tmem119-PE/Cy7 in BV2 cells and primary microglia. (**G**) Quantification of Tmem119-PE/Cy7 MFI in BV2 cells and primary microglia. *P* value is the result of unpaired two-tailed T-test. *N* = 3 replicates of BV2 cells and primary microglia from 3 mice. (**H**) Relative expression of *Tmem119* detected by RT-qPCR in BV2 cells and primary microglia. *P* value is the result of unpaired two-tailed T-test. *N* = 3 replicates of BV2 cells and primary microglia from 3 mice

Given the difficulty in culturing primary microglia in vitro, immortalized microglia-like cell lines are often used. BV2 cells are a murine microglia-like cell line which have been extensively used to study neuroinflammation [19]. In these experiments, we compared the expression of Tmem119 in BV2 cells to primary microglia. Previous studies have shown that LPS can induce an inflammatory response in BV2 cells which is similar to primary microglia [20, 21]. After treatment for 24 h with 5 μg/mL LPS, flow cytometry analysis was performed to detect Tmem119 expression (Fig. 3A). Tmem119 expression was detected on BV2 cells compared to isotype control (Fig. 3B and C). Unlike primary microglia, no change in Tmem119 expression could be detected in BV2 cells after LPS treatment (Fig. 3B and C). Moreover, LPS also did not induce *Tmem119* gene expression changes when measured by RT-qPCR (Fig. 3D). We next directly compared Tmem119 expression levels between BV2 cells and primary microglia (Fig. 3E). Compared to BV2 cells, primary microglia had a significantly higher expression of Tmem119 by flow cytometry (Fig. 3G) and RT-qPCR (Fig. 3H). In summary, BV2 cells have substantially lower Tmem119 expression at both the protein and gene expression levels compared to primary microglia. Furthermore, LPS was not able to induce the loss of Tmem119 expression which was observed in primary microglia.

### Tmem119 downregulation in brain metastasis-associated microglia

**Fig. 4:**
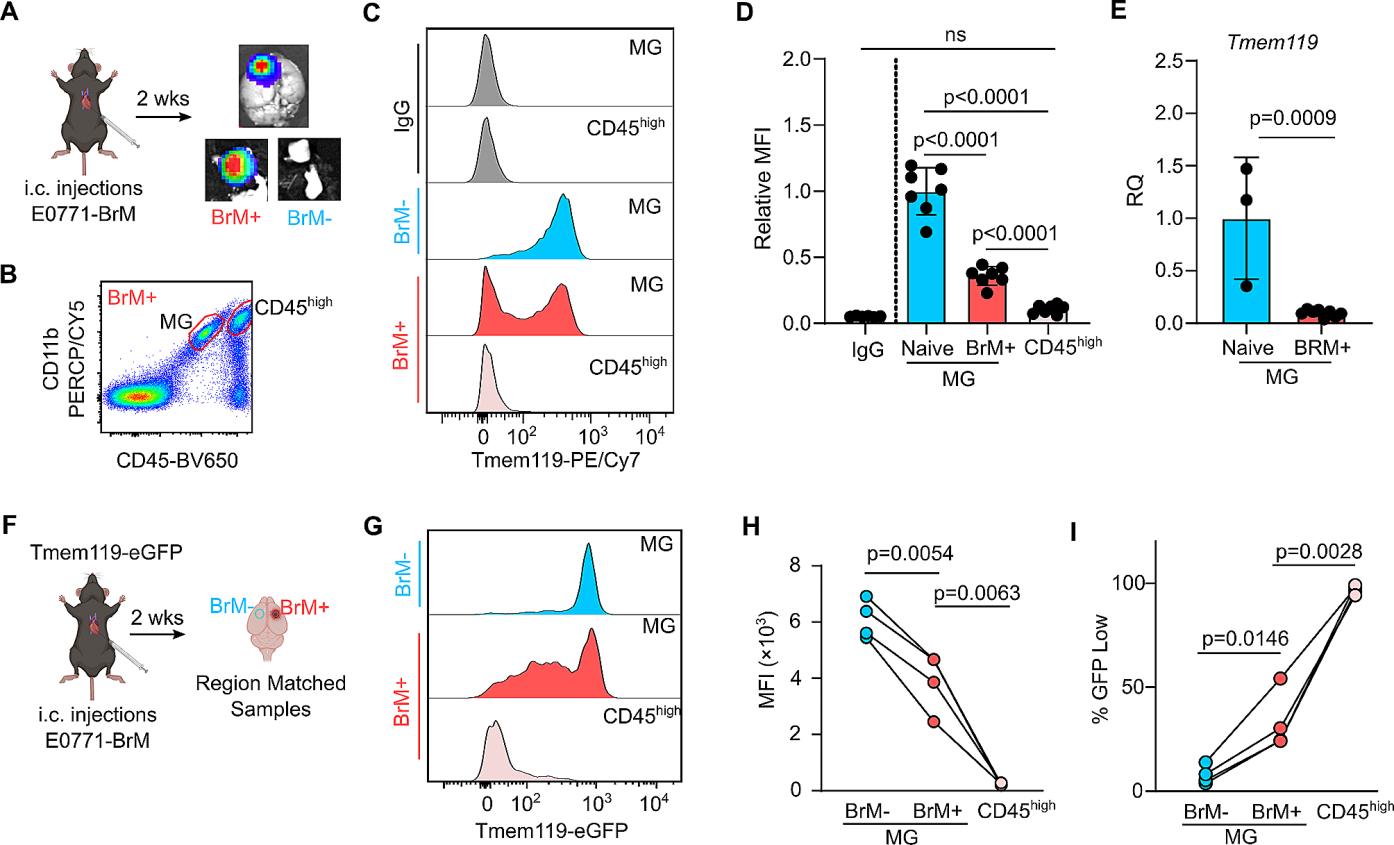
Microglia Tmem119 is downregulated in breast cancer brain metastasis. (**A**) Breast cancer brain metastases were established by intracardiac injection of E0771-BrM cells. Control tissue (BrM-) and tumor samples (BrM+) were collected based on ex vivo bioluminescence imaging. (**B**) Representative gating strategy used to identify microglia and infiltrating CD45^high^ myeloid cells in BrM + samples. (**C**) Representative flow cytometry histogram of Tmem119-PE/Cy7 and isotype controls. (**D**) Quantification of Tmem119-PE/Cy7 MFI in the different cell populations. *P* values are the result of one-way ANOVA with Tukey HSD. Data are presented as mean ± S.D. *N* = 7 mice. (**E**) RT-qPCR analysis comparing relative quantities of *Tmem119* in microglia sorted from naïve mice brains and BrM + samples. *P* values are the results of unpaired two-tailed T-test. Data are presented as mean ± S.D. *N* = 3 naïve mice and 8 BrM + samples. (**F**) Brain metastases were established in Tmem119-eGFP mice, and paired BrM- samples were collected from anatomically matched brain regions on the non-tumor-bearing hemisphere of the same animal. (**G**) Representative flow cytometry histogram of Tmem119-eGFP signal from BrM- MG, BrM + MG, and BrM + CD45^high^ myeloid cells. (**H**) Quantification of Tmem119-eGFP MFI in the different cell populations. *P* values are the results of paired two-tailed T-tests. *N* = 4 mice. (**I**) Percentage of GFP^low^ populations in microglia and CD45^high^ myeloid cells. *P* values are the results of paired two-tailed T-tests. *N* = 4 mice

Lastly, we employed a syngeneic mouse model of breast cancer brain metastasis (E0771-BrM) to investigate Tmem119 expression in tumor-associated microglia. The E0771-BrM cells were produced by 3 rounds of in vivo selection to obtain highly brain-tropic cells [22]. The cells are transduced to express far-red luciferase to facilitate identification of tumors by ex vivo bioluminescence imaging. Brain metastases were established by intracardiac injections. From the mice with developed tumors, brain metastatic lesions (BrM+) and control tissue (BrM-) were collected based on ex vivo bioluminescence signals (Fig. 4A). The samples were dissociated into single cell suspensions and Tmem119 expression was assessed by flow cytometry analysis. In the BrM + samples, microglia were gated on the CD45^int^CD11b^pos^ population while infiltrating myeloid cells were gated on the CD45^high^CD11b^pos^ population (Fig. 4B).

Initially, we used the Tmem119-PE/Cy7 antibody-based approach to compare expression levels in BrM- and BrM + samples (Fig. 4C). Similar to LPS-activated microglia, Tmem119 MFI was significantly reduced in BrM + microglia compared to BrM- microglia (Fig. 4D). In agreement with the existing literature, CD45^high^ infiltrating myeloid cells do not appear to express Tmem119 (Fig. 4C and D). When we sorted BrM + microglia and compared *Tmem119* gene expression levels to naïve microglia, there was also a significant reduction in mRNA levels (Fig. 4E). However, when observing the histogram profile (Fig. 4C), the BrM + microglia with reduced Tmem119 expression were virtually indistinguishable from infiltrating myeloid cells. Thus, using the Tmem119 antibody would not be able to reliably separate these two populations.

We then established E0771-BrM tumors in the Tmem119-eGFP transgenic mice. In these experiments, we took matched BrM- control samples from the same anatomical regions on the opposite non-tumor-bearing hemisphere in each animal with developed tumors (Fig. 4F). Again, we detected a loss of Tmem119 expression in BrM + microglia compared to BrM microglia (Fig. 4G and H). CD45^high^ infiltrating myeloids had the lowest Tmem119-eGFP MFI (Fig. 4H). There was a significant increase in GFP^low^ microglia in BrM + compared to BrM- samples, suggesting that a subset of microglia have reduced Tmem119 expression in the tumors (Fig. 4I). Importantly, nearly all (> 99%) of the infiltrating CD45^high^ population were GFP^low^, suggesting that Tmem119 is microglia-specific and is not expressed on tumor-infiltrating peripheral myeloid cells. Finally, unlike the Tmem119 antibody-based detection, BrM + microglia with reduced Tmem119 could still be identified by flow cytometry to separate this population from CD45^high^ infiltrating myeloid cells (Fig. 4C and G). Altogether, we conclude that a subpopulation of BrM + microglia have reduced Tmem119 expression in the brain metastatic tumor microenvironment.

## Discussion

In agreement with previous studies, we detected a strong Tmem119 expression in homeostatic microglia in vivo (Fig. 1B,C) [8]. Furthermore, Tmem119 immunoreactivity was significantly reduced after activation by LPS and brain metastases [11, 13, 23, 24]. After activation, microglia undergo morphological changes and adopt an amoeboid shape which could explain the loss of Tmem119 surface protein expression. However, one study reported that the change to microglia morphology was not congruent with loss of Tmem119 expression in a murine model of ischemic stroke [12]. In our study, we further show that overall *Tmem119* mRNA is significantly reduced in microglia after LPS treatment (Fig. 2D) and in the brain metastatic lesions (Fig. 4E). Therefore, a reduction in Tmem119 expression could be a potential marker for microglia activation state.

Microglia are known to have differing transcriptomic profiles based on the brain region [16, 17, 25]. A recent study by Barko *et al.* reports that midbrain microglia have a gene signature enriched in immune-related pathways, suggesting a more immune-vigilante state [17]. In their dataset, midbrain microglia *Tmem119* expression is the lowest compared to prefrontal cortex and striatum microglia. While their dataset did not include cerebellum, other studies have reported that cerebellar microglia are also in a heightened immune-vigilante state [25–27]. In our study, we detected a substantial amount of GFP^low^ microglia in the cerebellum even without LPS treatment (Fig. 2G). Cerebellar microglia is reported to lose Tmem119 expression during aging, along with a significant shift in their transcriptome compared to young homeostatic microglia [25]. Cerebellar microglia also have a less ramified morphology and higher turn-over rate compared to cortical microglia [26, 27]. Several other groups have shown a reduction in *Tmem119* gene expression in neurodegeneration and tumor-associated microglia [4, 6, 28–30]. Overall, these studies support a link between loss of Tmem119 expression and microglia activation state.

Microglia are traditionally identified by CD45^int^ staining, but there are some concerns regarding its use in a disease-related context. Activated microglia are reported to increase CD45 immunoreactivity which would decrease its reliability as a marker to distinguish these cells from infiltrating CD45^high^ cells [31]. Indeed, we also observed a more variable CD45 expression in the microglia from brain metastatic lesions. However, the change in CD45 immunoreactivity was not significant enough to overlap with infiltrating myeloid cells (Fig. 4B). Nevertheless, a combination with other markers such as Tmem119 will help strengthen flow cytometric analyses and allow sorting a purer microglia population.

It is important to note that some studies showed induction of microglia-specific markers, including Tmem119, in recruited peripheral myeloids by the brain microenvironment [32, 33]. However, we did not detect any Tmem119 expression in CD45^high^ infiltrating myeloid cells in our brain metastasis model (Fig. 4C-D,G-H). Of note, when using the Tmem119 antibody a subpopulation of BrM-associated microglia appears to completely lose Tmem119 expression, to the point of having the same expression levels as CD45^high^ infiltrating myeloid cells (Fig. 4C). It has been reported that the extracellular domain of Tmem119 can be cleaved during activation, resulting in loss of antibody binding [23]. Using the Tmem119-eGFP transgenic mice, we show that while Tmem119 expression in BrM + microglia is still decreased, it remains clearly higher than the overall signal from CD45^high^ cells. Therefore, it is possible to distinguish Tmem119^low^ BrM-associated microglia from infiltrating CD45^high^ myeloid cells using these transgenic mice (Fig. 4G). More importantly, this will allow sorting of Tmem119^high^ and Tmem119^low^ microglia to investigate and better define the role of these cells in brain metastasis progression in future studies. Similar to peripheral macrophages, microglia can have a diverse array of polarization states ranging from pro-inflammatory (M1) to anti-inflammatory (M2) [34]. While this study did not directly link loss of Tmem119 to any particular microglia phenotype, we speculate that loss of Tmem119 would be associated with changes in immune function. While the anti-inflammatory (M2-like) phenotype is generally associated with tumor progression, recent single cell sequencing studies show that tumor-associated microglia actually have a more pro-inflammatory (M1-like) signature while the immune-suppressive gene signatures are enriched in infiltrating macrophages [4, 5, 30]. As a result of these emerging differences between microglia and macrophages, there has been a general suggestion in the field to move away from the classical M1/M2 characterization of microglia in order to truly appreciate their complexity [35–38].

For flow cytometry, the method of tissue digestion and preparation of single cell suspensions is very important. The data from our experiments used a combination of type III collagenase and physical dissociation to prepare cell suspensions from brains and brain tumor samples. Of note, we also tested another digestion method with the commonly used papain enzyme. Papain is a non-specific protease which has been shown to be one of the gentlest methods in preparing cell suspensions from brain tissue [39–41]. It is also commercially available as the main component of neural tissue and brain tumor digestion kits. In our samples prepared with papain, we were unable to detect Tmem119 by flow cytometry using the antibody-based approach, likely due to epitope degradation (data not shown). The use of the Tmem119-eGFP transgenic mice could overcome these limitations.

Lastly, we found that the immortalized BV2 microglia cell line had a low basal expression of Tmem119. Using both flow cytometry and RT-qPCR, our results indicate that the BV2 cells have a significantly reduced expression of Tmem119 compared to the in vivo microglia (Fig. 3F-H). Furthermore, treatment of BV2 with LPS was not able to induce a further decrease in Tmem119 expression (Fig. 3B-D). It is possible that BV2 cells are already in a partially activated state due to immortalization and in vitro culture conditions. Previous studies using the BV2 cell line have reported a dampened immune response compared to primary microglia [19, 42, 43]. Therefore, one should be aware of any limitations in using immortalized microglia-like cell lines compared to primary microglia.

## Conclusion

In conclusion, our study confirms that Tmem119 is a robust marker for homeostatic microglia. We used an LPS and breast cancer brain metastasis model to show a reduction in Tmem119 gene and protein expression after pathological activation. The microglia-like BV2 cell line was not able to recapitulate Tmem119 expression patterns observed with primary microglia. Finally, we validated the use of the Tmem119-eGFP transgenic mice and found a distinct population of Tmem119^low^ microglia in brain metastatic tumors.

## Materials and methods

### Animal experiments

All animal experiments were performed in accordance with protocols and guidelines approved by the Wistar Institutional Animal Care and Use Committee, The Association for Assessment and Accreditation of Laboratory Animal Care, and the NIH Office of Laboratory Animal Welfare. All animals were euthanized at appropriate experimental or humane endpoints. Humane endpoints include loss of motor function, moribund state, or loss of 20% total body weight. Animals were housed in temperature and humidity-controlled environments on a 12-hour light-dark cycle. Potential confounders such as order of measurements and animal/cage location were randomized. Wild-type C57BL/6J and Tmem119^em2Gfng^/J (Tmem119-eGFP) mice were purchased from The Jackson Laboratory. Both male and female mice between 6 and 8 weeks of age were used for the LPS experiments. Female mice were used for the breast cancer brain metastasis experiments. Mice were randomly placed into experimental groups. For LPS treatment, mice received 5 mg/kg intraperitoneal injections 24 h before euthanasia. Brain metastases were established by injecting 2.5 × 10^4^ E0771-BrM cells into the left cardiac ventricle. Mice that did not develop brain metastases were excluded from the analysis. For mice treated with LPS, euthanasia was performed by CO_2_ inhalation until loss of vital signs were observed. Brain tissues were then harvested and collected in ice cold PBS. For identification of BrM + lesions by ex vivo bioluminescence imaging, mice were anesthetized by inhalation of 2–4% isoflurane. 40 mg/kg luciferin was administered by retro-orbital injections immediately before euthanasia by cervical dislocation. Brains were removed and imaged on an IVIS Spectrum CT.

### Cell culture

BV2 cells were kindly provided by the lab of Dr. Dario Altieri at The Wistar Institute. BV2 cells were cultured in DMEM containing 10% FBS and 1× GlutaMAX supplement. Cells were maintained under standard incubation conditions (37 °C, 5% CO_2_). Cells were routinely subcultured upon reaching 80% confluency. Mycoplasma testing was performed every 6 months using the Lonza MycoAlert Kit (LT07-318). For LPS experiments, cells were plated at 2 × 10^5^ cells/well in 6-well plates and treated for 24 h with 5 μg/mL LPS.

### Flow cytometry and cell sorting

Harvested brain tissue were digested in 200 U/mL collagenase III solution (Worthington CLS-3). After 10 min incubation at 37 °C, samples were mechanically dissociated using a P1000 pipette and incubated an additional 10 min. BV2 cells were collected using a sterile cell scraper. Equal volume cold PBS was added, and the samples were filtered through a 70 μm cell strainer. Samples were centrifuged at 200×*g* for 5 min. After discarding supernatant, samples were resuspended in FACS buffer (PBS containing 2.5% BSA and 2 mM EDTA) containing 1:100 CD16/32 FcR blocking antibody (BD553143) and incubated at 4 °C for 10 min. Next an antibody cocktail containing Zombie Aqua (BioLegend 423,101), CD45-BV650 (BioLegend 1,031,515), CD11b-PerCP/Cy5.5 (BioLegend 101,227), and Tmem119-PE/Cy7 (Invitrogen 25-6119-82) were added at a 1:100 final dilution for all components. The samples were incubated at 4 °C in the dark for an additional 20 min. Samples were washed twice with FACS buffer and data were acquired on a BD LSRII flow cytometer. The CD45^int^CD11b^pos^ population were sorted on a BD FACSymphony S6.

### RNA extraction and RT-qPCR

RNA was purified from sorted microglia and BV2 cells with the Zymo Research Quick-RNA Microprep Kit (R1050) following the manufacturer’s protocol. The RNA was reverse transcribed into cDNA using the ThermoFisher RevertAid RT Kit (K1691) following the manufacturer’s protocol. RT-qPCR was performed using the PowerUp SYBR Green Master Mix (Applied Biosystems A25742) on the QuantStudio 6 and 7 Flex Real-Time PCR systems. Primer sequences used: *Tmem119* (Forward: CGGCCTATTACCCATCGTCC, Reverse: CTGGGCTAACAAGAGAGACCC). *Actb* (Forward: GGCTGTATTCCCCTCCATCG, Reverse: CCAGTTGGTAACAATGCCATGT).

### Immunofluorescence staining

Whole brains were collected from mice and fixed by overnight incubation at 4 °C in 4% paraformaldehyde. Brains were sequentially washed and incubated overnight at 4 °C in PBS, 15% D-Sucrose, and 30% D-Sucrose solutions. 80 μm sections were cut along the rostral-caudal axis on a cryotome. Sections were collected in PBS containing 30% PEG300 (Sigma-Aldrich 807,484) and 30% glycerol (Invitrogen 15,514,011) and were stored at -20 °C until staining. Sections were transferred into 24-well plates and washed with PBS three times to remove antifreeze media. Sections were blocked and permeabilized in PBS containing 10% normal goat serum (ThermoFisher 50197Z), 0.25% Triton-X100 (Sigma-Aldrich X100), and 2% BSA (Roche 03117332001) for 2 h. Chicken anti-GFP primary antibody (Aves Labs GFP-1020) was prepared at 1:1000 dilution in the blocking solution and incubated with the samples overnight at 4 °C. Samples were washed with 0.25% Triton-X100 in PBS three times. Goat anti-chicken AlexaFluor 488 was added at 1:500 dilution and samples were incubated in the dark for 2 h. After washing with PBS three times, sections were mounted onto slides for imaging on a Leica TCS SP5 laser confocal microscope. Quantification of GFP staining intensity was performed in the ImageJ software. Individual 8-bit images were duplicated, and a threshold was applied to the duplicated image (min 50, max 255). The Analyze Particles function was used to draw ROIs around individual microglia based on the threshold image (size limit = 25 to inf). GFP intensity was calculated from ROIs overlaid on the original unmodified images.

### Statistical analysis

All statistical analyses were performed in GraphPad Prism Version 8. Data are presented as mean ± standard deviation. Experimental groups were not blinded. For experiments comparing two independent groups, an unpaired two-tailed Student’s t-test was performed with alpha = 0.05. For matched BrM + and BrM- samples, a paired two-tailed Student’s t-test was performed with alpha = 0.05. For comparison of multiple groups, *p* values were calculated by one-way ANOVA and Tukey Post Hoc test. For animal experiments, sample sizes were estimated based on previous experiments using these models calculated to achieve 80% power with alpha = 0.05.

## Data Availability

All datasets used and/or analyzed during the current study are available from the corresponding authors upon reasonable request.
